# The Fellowship of the Royal College of Surgeons

**Published:** 1909-09-04

**Authors:** 


### THE FELLOWSHIP OF THE ROYAL COLLEGE OF SURGEONS.

The Fellowship of the Royal College of Surgeons of England is justly esteemed as the premier qualification that the student can obtain in surgery, and is almost an essential to any man who aspires to the higher surgical posts in England. The student should decide early in his career whether he wishes to obtain the F.R.C.S. For this purpose it is necessary to pass two examinations, a preliminary and a final.

The Primary examination takes place twice every year, in May and in November, and is held in London. The subjects are Anatomy and Physiology, and candidates must have passed the second Conjoint examination or some other equivalent examination which exempts them from it. They must also (except in the case of qualified men) have spent two winter sessions in dissections and attendance at physiology classes at a recognised school. The examination is partly oral and partly written. Two papers are set, one in physiology and one in anatomy, four questions being given with an allowance of three hours. The oral examinations are two, lasting fifteen minutes each, in the two subjects. The examination has the reputation of being unusually stiff, with more than the average element of luck appertaining to it, but its difficulty should not frighten the student or practitioner of reasonable ability who is willing to pay special attention to the two subjects. A good, sound knowledge of anatomy and physiology is required, and if the candidate has been diligent at his practical work in the dissecting room and laboratory, and has supplemented the knowledge there gained by reading, he stands a good chance of success.

The examination for the Final Fellowship takes place in May and November of each year, and consists of written papers, *viva voces*, clinical examinations, and operative surgery. The fee for the examination is £15 15s., and candidates are required to be members of the college who have been six years in practice or in the study of medicine. Special classes for the Final Fellowship examination are held at most of the London hospitals; and practitioners who intend to enter for it, and have lost touch to any extent with hospital surgical practice, would do well to attend a full course of instruction at one of the medical schools before competing.

